# Vitamin D_3_ and Dimethyl Fumarate Partially Restore Neurotrophic Signaling Without Altering Mitochondrial Integrity in the STZ-Induced Model of Sporadic AD

**DOI:** 10.3390/ijms27114940

**Published:** 2026-05-29

**Authors:** Natalia Piekarczyk, Paweł Berezka, Kalina Domkowicz, Dorota Myślińska, Jan Jacek Kaczor

**Affiliations:** Department of Animal and Human Physiology, Faculty of Biology, University of Gdansk, 80-309 Gdansk, Poland; natalia.piekarczyk@ug.edu.pl (N.P.); pawel.berezka@ug.edu.pl (P.B.); dorota.myslinska@ug.edu.pl (D.M.)

**Keywords:** sporadic Alzheimer’s disease, vitamin D_3_, dimethyl fumarate, BDNF, Akt signaling, neuroprotection, redox imbalance

## Abstract

Alzheimer’s disease (AD) is characterized by impaired neurotrophic support, oxidative stress, and metabolic dysfunction. Using the intracerebroventricular streptozotocin (ICV-STZ) rat model of sporadic AD, we investigated whether vitamin D_3_ (VitD_3_) and dimethyl fumarate (DMF), administered alone or in combination, modulate hippocampal neurotrophin-related signaling and redox balance. Animals were assigned to SHAM, STZ, VITD, DMF, and COMBO groups, representing control, ICV-STZ, VitD_3_-treated ICV-STZ, DMF-treated ICV-STZ, and combined VitD_3_ + DMF-treated ICV-STZ animals, respectively. Hippocampal neurotrophin processing (proBDNF and mature BDNF), downstream signaling (Akt and pAkt), IGF-1 content, mitochondrial oxoglutarate dehydrogenase (OGDH) content, citrate synthase (CS) activity, and glutathione peroxidase (GPx) activity were assessed. STZ administration showed a trend toward reduced mature BDNF content compared with the SHAM group (*p* = 0.07), whereas combined VitD_3_ and DMF treatment significantly increased mature BDNF content compared with the STZ group. The mature BDNF/proBDNF ratio was reduced in the STZ group compared with the SHAM group and tended to be higher in the COMBO group compared with the STZ group (*p* = 0.09). proBDNF content remained unchanged. IGF-1, pTrkB, total Akt, and pAkt content did not differ significantly between groups. The pAkt/Akt ratio showed a trend toward reduction in the STZ group compared with SHAM group (*p* = 0.09). GPx activity increased in the STZ group, while CS activity and OGDH content were not significantly altered. These findings indicate that STZ-induced neurodegeneration is characterized by redox-associated uncoupling of neurotrophic signaling rather than mitochondrial disruption. Combined VitD_3_ and DMF treatment partially modulated neurotrophic signaling, supporting a limited but measurable neuroprotective effect.

## 1. Introduction

Alzheimer’s disease (AD) is a progressive neurodegenerative disorder characterized by cognitive decline, synaptic dysfunction, and disturbances in metabolic and redox homeostasis [1,2]. Increasing evidence suggests that sporadic forms of AD are closely associated with impaired brain insulin signaling, oxidative stress, and neuroinflammation [3]. Moreover, sporadic AD (sAD) lacks a clear genetic basis and is strongly associated with neuroinflammation, although its precise molecular mechanisms remain unclear. Intracerebroventricular (ICV) administration of streptozotocin (STZ) is a widely used experimental model that mimics key features of sAD, including brain insulin resistance, cognitive impairment, disturbances in oxidative balance, neuroinflammation, and neuronal dysfunction, making it a valuable model for investigating disease-related pathophysiological mechanisms [4,5,6]. Consistently, STZ-induced neurodegeneration has been linked to increased oxidative stress, mitochondrial dysfunction, and impairment of neurotrophic signaling pathways, including BDNF- and IGF-related mechanisms. These alterations contribute to synaptic failure and progressive cognitive decline, highlighting the importance of metabolic and redox regulation in AD pathogenesis [7,8,9]. In recent years, increasing attention has been directed toward the role of vitamin D as a modulator of brain function. Vitamin D acts as a neuroactive steroid, exerting its effects through the vitamin D receptor (VDR), which is widely expressed in neurons and glial cells. A growing body of evidence indicates that vitamin D is involved in neuroprotection, regulation of neurotransmission, and maintenance of redox balance in the central nervous system [10,11]. Importantly, studies have shown that vitamin D_3_ treatment may influence mitochondrial function, oxidative stress parameters, and signaling pathways associated with neuronal survival, including brain-derived neurotrophic factor (BDNF) and protein kinase B (Akt)-related mechanisms [12]. In the ICV-STZ model, vitamin D_3_ was shown to ameliorate memory dysfunction, at least in part, through protection of the antioxidant and cholinergic systems [13]. These findings suggest that vitamin D may modulate pathways relevant to neurodegeneration. Another compound of interest is dimethyl fumarate (DMF), a molecule with established anti-inflammatory and antioxidant properties. DMF exerts its effects primarily through activation of the nuclear factor erythroid 2-related factor 2 (NRF2) pathway, leading to the upregulation of cytoprotective and antioxidant responses. This mechanism has been implicated in neuroprotection and mitigation of oxidative damage in various models of neurodegeneration [14,15].

Given that the ICV-STZ model involves overlapping disturbances in insulin-related signaling, oxidative stress, neuroinflammation, and neurotrophic regulation [4,5,6,7,8,9], a combined intervention may be relevant when the tested compounds target complementary aspects of this pathology. The rationale for combining VitD_3_ and DMF was based on the possible convergence between neurotrophic/Akt-related signaling and NRF2-dependent cytoprotective responses, rather than on a single shared mechanism [10,11,12,13,14]. This provided a basis for examining whether simultaneous modulation of these pathways may result in additive or partially synergistic effects in the STZ-induced sAD model. This rationale is further supported by our previous study, in which combined VitD_3_ and DMF treatment produced the most consistent behavioral improvement and partially attenuated oxidative and inflammatory alterations in the same experimental model [16]. Therefore, the present study aimed to investigate whether combined VitD_3_ and DMF treatment modulates neurotrophin-related signaling, with particular emphasis on BDNF maturation and downstream Akt signaling, as well as redox balance, in the STZ-induced sAD model.

## 2. Results

### 2.1. Combined VitD_3_ + DMF Treatment Modulates Neurotrophin-Related Signaling

**Figure 1:**
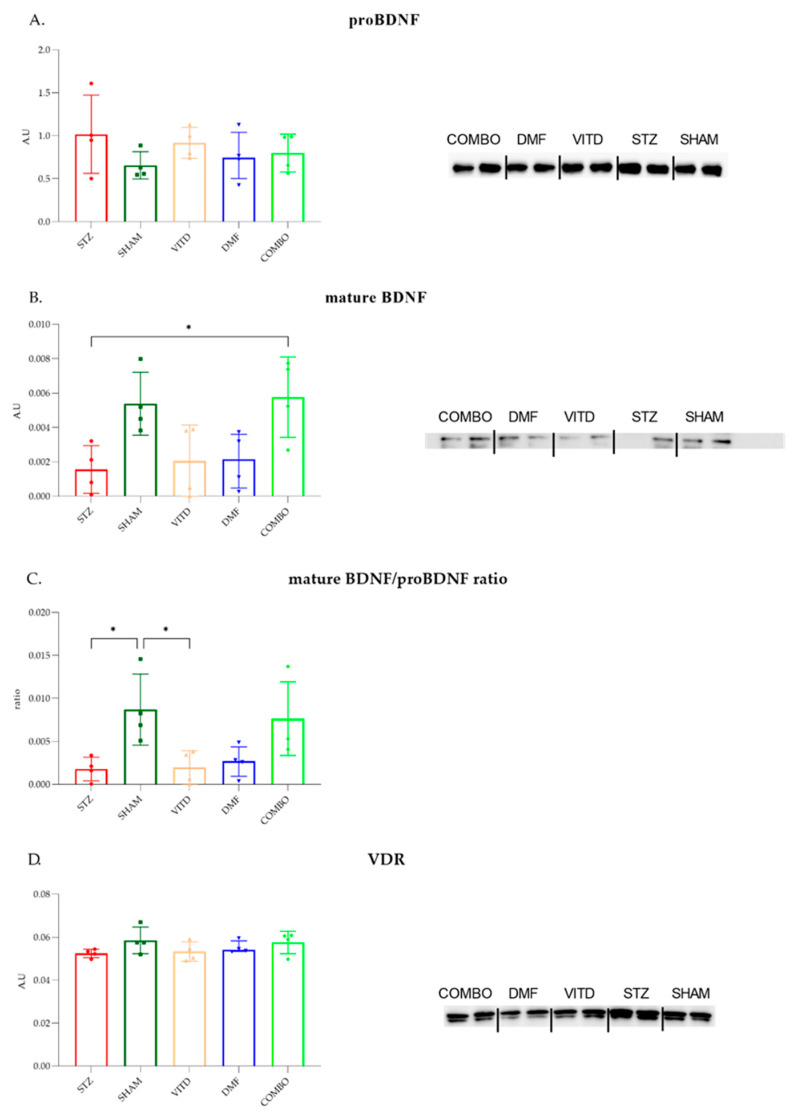
Neurotrophin balance and VDR content in the hippocampus of ICV-STZ rats. (**A**) proBDNF protein content, (**B**) mature BDNF protein content, (**C**) mature BDNF/proBDNF ratio, and (**D**) protein content of VDR in the experimental groups. Representative immunoblots are shown next to the corresponding quantitative analyses. All proteins were normalized to the total protein load using stain-free technology. Data are presented as mean ± SD, with individual data points shown * *p* < 0.05.

To evaluate the effect of combined administration with VitD_3_ and DMF on the neurotrophin-related signaling pathway, we analyzed BDNF content in hippocampal supernatants. The proBDNF protein content (Figure 1A) did not differ between groups. Mature BDNF content showed a trend toward reduction in the STZ group compared with the SHAM group (*p* = 0.07; Figure 1B). Combined with VitD_3_ and DMF treatment significantly increased mature BDNF content compared with the untreated STZ group (*p* < 0.05; Figure 1B), whereas monotherapy treatments did not differ significantly from the STZ group (Figure 1B). Accordingly, the mature BDNF/proBDNF ratio was significantly reduced in the STZ group compared with the SHAM group and tended to increase following combined treatment, further supporting impaired BDNF maturation and its modulation in the COMBO group (*p* < 0.05; *p* = 0.09; Figure 1C). Notably, the lack of changes in proBDNF suggests that the observed effect is primarily related to impaired BDNF maturation rather than altered precursor availability. VDR content did not differ significantly between groups (Figure 1D), indicating that the observed effects are unlikely to be mediated by changes in receptor abundance.

### 2.2. The Role of IGF-1 and pTrkB in the Experimental Group

**Figure 2:**
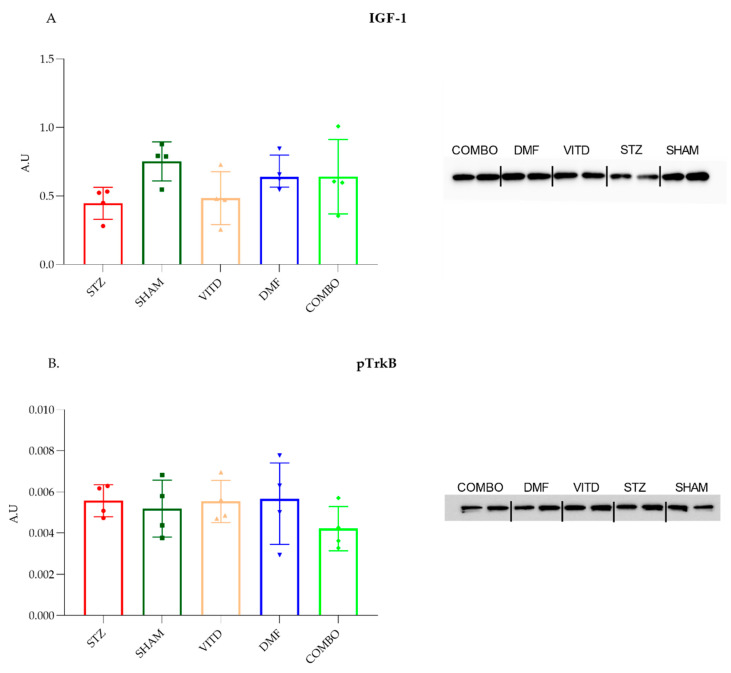
IGF-1 and pTrkB content in the hippocampus of ICV-STZ rats. (**A**) Insulin-like growth factor 1 (IGF-1) protein content and (**B**) phosphorylated tropomyosin receptor kinase B (pTrkB, Tyr816) content in the experimental groups. Representative immunoblots are shown next to the corresponding quantitative analyses. All proteins were normalized to the total protein load using stain-free technology. Data are presented as mean ± SD, with individual data points shown.

To evaluate the effects of administration with VitD_3_ and DMF on neurotrophin-related signaling pathways, we measured IGF-1 and pTrkB content in the hippocampus supernatants. IGF-1 protein content showed a lower mean value in the STZ group compared with the SHAM group, whereas higher mean values were observed in the treatment groups; however, these differences did not reach statistical significance (Figure 2A). No significant differences in pTrkB content were observed between groups (Figure 2B). Exploratory correlation analyses further indicated that IGF-1 showed directionally consistent associations with selected behavioral outcomes, providing additional context for its potential functional relevance (Figure S2).

### 2.3. Combined Treatment Affects Akt Signaling

**Figure 3:**
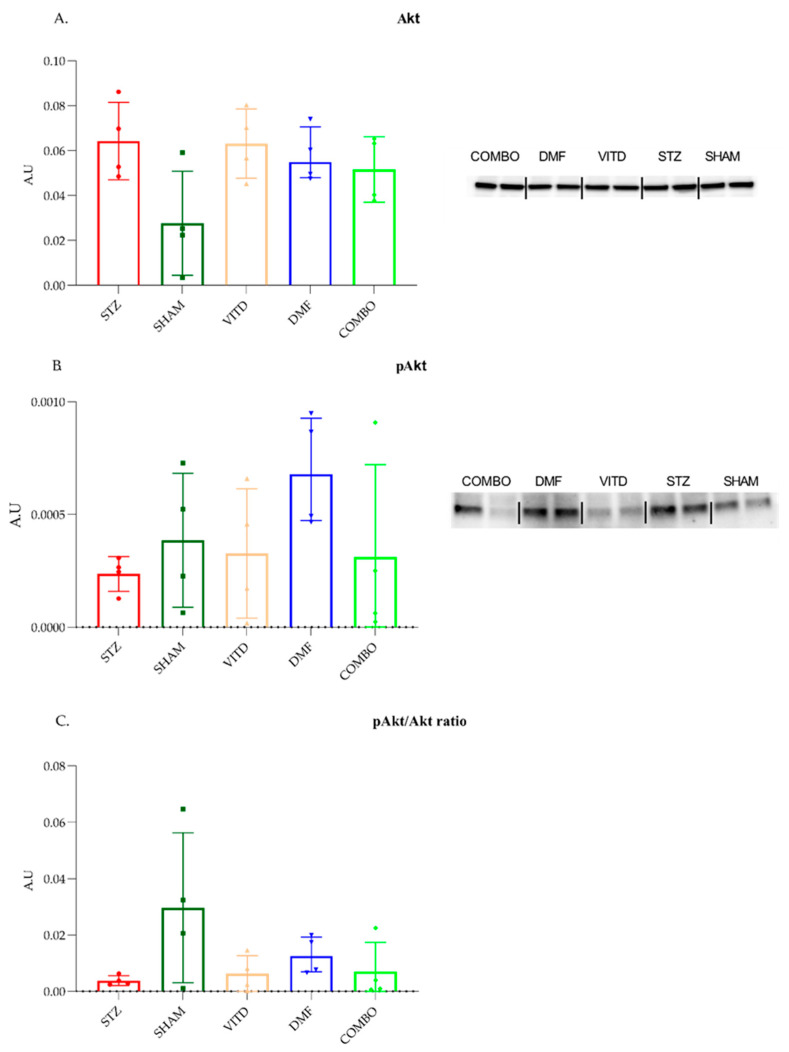
Akt signaling in the hippocampus of ICV-STZ rats. (**A**) Total Akt protein content, (**B**) phosphorylated Akt (pAkt, Thr308) content, and (**C**) pAkt/Akt ratio in the experimental groups. Representative immunoblots are shown next to the corresponding quantitative analyses. Total Akt and pAkt were normalized to the total protein load using stain-free technology. The pAkt/Akt ratio was calculated using stain-free-normalized values. Data are presented as mean ± SD, with individual data points shown.

To further investigate downstream signaling associated with neurotrophin modulation, we analyzed total Akt protein content, pAkt (Thr308) content, and the pAkt/Akt ratio. Total Akt content did not differ significantly between groups (Figure 3A,B). The pAkt/Akt ratio showed a trend toward reduction in the STZ group compared with the SHAM group (*p* = 0.09; Figure 3C), with no significant treatment-related differences detected. Together with the BDNF-related changes, these results suggest that STZ-induced alterations in neurotrophic signaling were not accompanied by robust activation of downstream Akt signaling at the analyzed time point.

### 2.4. Combined Treatment Affects Redox Balance Without Altering Selected Mitochondrial Enzymes 

**Figure 4:**
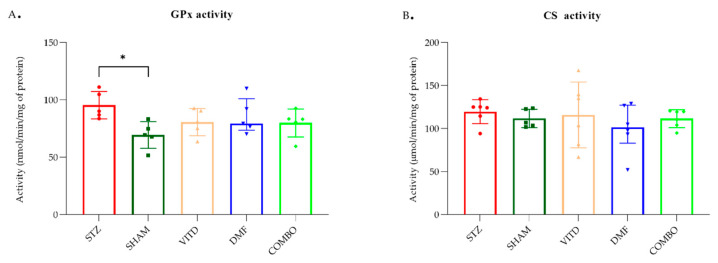
Metabolic context: GPx and CS activity in the hippocampus of ICV-STZ rats. (**A**) Glutathione peroxidase (GPx) and (**B**) citrate synthase (CS) activity in the experimental groups. Enzyme activity is expressed as nmol/min/mg of protein and µmol/min/mg of protein, respectively. Data are presented as mean ± SD, with individual data points shown * *p* < 0.05.

To assess whether changes in neurotrophic signaling were accompanied by alterations in oxidative and metabolic responses, GPx and CS activities, as well as OGDH protein content, were evaluated. GPx activity significantly increased in the STZ group compared with the SHAM group (*p* < 0.05; Figure 4A), indicating activation of antioxidant defense mechanisms. Treatment groups did not differ significantly from the untreated STZ group, although lower mean values of GPx activity were observed in the VITD and COMBO groups. In contrast, CS activity (Figure 4B) did not differ between groups, suggesting no detectable changes in this marker of mitochondrial enzymatic capacity. Similarly, OGDH protein content showed no significant differences (Figure S1). Together, these findings indicate that redox-related alterations were detectable in the STZ model, whereas they were not accompanied by significant changes in the selected mitochondrial enzyme markers assessed in this study.

## 3. Discussion

The present study demonstrates that STZ-induced neurodegeneration is associated with alterations in neurotrophin-related parameters, including BDNF, together with no significant changes in VDR content. In particular, mature BDNF content showed a trend toward reduction in the STZ group and its increase following combined treatment was consistent with impaired neurotrophic support in this model [17,18].

Importantly, in our previous report based on the same experimental paradigm, combined VitD_3_ and DMF administration improved cognitive performance and reduced oxidative stress and inflammatory markers [16]. The present findings extend these observations by showing that BDNF-related parameters may accompany the previously observed behavioral improvement. Although no causal relationship can be established, this pattern is consistent with a shift toward a more favorable neurotrophic environment. In line with this interpretation, exploratory correlation analyses provided additional context by showing associations with directions consistent with selected neurotrophic-related endpoints and behavioral outcomes. Given the limited number of matched samples, these findings should be considered supportive rather than conclusive (Figure S2). The observed changes in mature BDNF content and in the mature BDNF/proBDNF ratio may also be interpreted in the context of BDNF processing. proBDNF may be converted to mature BDNF through intracellular cleavage by furin/proprotein convertases and extracellular cleavage by proteases such as plasmin and matrix metalloproteinases [19]. Thus, the reduced mature BDNF/proBDNF ratio observed in the STZ group may reflect altered BDNF maturation rather than reduced precursor availability alone. Since these proteolytic processes are part of the regulatory environment of neurotrophin signaling, the redox- and inflammation-modulating actions of VitD_3_ and DMF described previously [18] may provide a plausible context for the partial modulation of BDNF-related parameters in the COMBO group. However, this mechanism was not directly assessed in the current study.

No significant differences in VDR content were observed between groups. Given that vitamin D signaling has been linked to BDNF/Akt-related pathways, mitochondrial homeostasis, and antioxidant capacity [12,20], these findings indicates that the BDNF-related effects observed in the present study were not accompanied by detectable changes in the VDR protein content. In our previous study, combined VitD_3_ and DMF treatment altered circulating vitamin D metabolites, showing increased ligand availability [18]. Thus, the lack of changes in VDR content does not exclude a vitamin D-related contribution to the observed molecular profile, but indicates that this contribution was not reflected at the level of total receptor abundance. Moreover, as vitamin D signaling may include rapid non-genomic components, the single terminal time point used in the present study may not fully capture transient changes in downstream signaling [11].

In this study, STZ administration was associated with lower IGF-1 content, whereas higher mean values were observed in the treated groups; however, these differences did not reach statistical significance. Given the role of IGF-1 in neuronal survival, synaptic function, and metabolic regulation, these descriptive findings place IGF-1 within the broader neurotrophic–metabolic context of STZ model [21,22]. In addition, exploratory correlation analyses showed directionally consistent associations between IGF-1 and selected behavioral outcomes, further supporting its potential functional relevance. Disruption of insulin and IGF-related pathways is a recognized feature of this model and has been linked to cognitive impairment and neuronal dysfunction. Although IGF-1 content did not differ significantly between groups, its lower mean value after STZ administration, higher mean values in treated animals, and directionally consistent exploratory correlations with behavioral outcomes support its relevance to the neurotrophic profile observed in this model. The lack of a significant treatment-related change may reflect the fact that IGF-related signaling depends not only on ligand content but also on receptor availability, IGF-binding proteins, and downstream pathway activation, which were not comprehensively assessed in the present study [23]. Despite alterations in neurotrophin-related parameters, no significant changes were observed in TrkB phosphorylation. This suggests that treatment effects may not involve robust activation of receptor-mediated signaling but rather modulation of upstream factors, such as ligand availability or metabolic context. The lack of significant changes in TrkB phosphorylation despite altered BDNF content may further support the notion of impaired receptor responsiveness or redox-dependent uncoupling of ligand–receptor signaling. The dissociation between changes in neurotrophin-related parameters and receptor activation may reflect the complexity of signaling regulation in neurodegenerative conditions, in which temporal dynamics may influence signaling outcomes. Overall, these findings suggest that neurotrophic-related signaling may be disrupted in the STZ group, although under the current experimental conditions, no significant differences in TrkB phosphorylation at Tyr816 were observed.

To further investigate downstream signaling, Akt protein content and activation were assessed. In the present study, total Akt and pAkt content did not differ significantly between groups, while the pAkt/Akt ratio showed a trend toward reduction in the STZ group compared with the SHAM group. No significant treatment-related differences were detected. Thus, the BDNF-related changes observed in the present study were not accompanied by robust alterations in Akt abundance or phosphorylation at the analyzed time point. Given the involvement of Akt in IGF-1- and neurotrophin-related signaling, this may reflect the complexity and temporal dynamics of downstream pathway regulation rather than a direct correspondence between changes in neurotrophic parameters and Akt activation [24,25,26]. These findings are in line with the lack of significant changes in TrkB phosphorylation and may indicate that downstream signaling pathways were not robustly engaged at the analyzed time point.

The present study also provides insight into the redox and metabolic context of STZ-induced neurodegeneration. The increase in GPx activity observed in the STZ group likely reflects a compensatory response to elevated oxidative stress, a well-established feature of this model [27,28,29]. Our group recently reported that STZ is associated with disruption of glutathione homeostasis, as reflected by an increased GSSG/GSH ratio, which was partially reversed by treatment [16]. Given that GPx catalyzes the reduction in peroxides using glutathione as a substrate, the observed increase in GPx activity may indicate enhanced glutathione turnover under oxidative stress conditions [9,10]. In this context, the partial normalization of GPx activity following treatment may be consistent with reduced oxidative burden rather than suppression of antioxidant defenses.

CS activity remained unchanged, suggesting that mitochondrial enzymatic capacity remained relatively stable. Together with the lack of changes in OGDH content, these findings suggest that the observed mitochondrial enzyme profile is more likely related to functional redox adaptation rather than structural alterations of mitochondria. Therefore, STZ-induced neurodegeneration is associated with altered redox homeostasis, while combined VitD_3_ and DMF treatment showed a shift toward redox normalization without detectable changes in the selected mitochondrial enzyme. This study has several limitations that should be acknowledged. Although disturbances in neurotrophic signaling and redox balance were identified, the precise mechanisms linking these alterations to STZ-induced neurodegeneration remain unclear. In particular, while no significant changes in selected mitochondrial enzymes were observed, this does not exclude the presence of mitochondrial dysfunction, as only selected proteins were assessed. The study focused on selected components of neurotrophic and redox signaling without evaluating the full spectrum of related pathways, which may limit the interpretation of the observed effects. In addition, the relatively small sample size may reduce the ability to detect subtle changes and limit the generalizability of the findings. Another important limitation is the use of only male rats, which may affect the translational interpretation of the results. The analysis was performed at a single experimental time point, precluding assessment of the temporal dynamics of signaling alterations. Moreover, the observed neuroprotective effects of combined VitD_3_ and DMF treatment were only partial, suggesting that this intervention may not fully counteract the complex pathological processes induced by STZ. Therefore, further studies are needed to examine additional signaling pathways, conduct a more comprehensive assessment of mitochondrial function, and determine whether the observed molecular changes are associated with long-term functional improvement.

Our findings suggest a mismatch between neurotrophins availability and downstream signaling activation, supporting the concept of functional uncoupling within neurotrophic pathways. This dissociation may result, at least in part, from redox imbalances rather than structural disruption to the mitochondria.

## 4. Materials and Methods

### 4.1. Animals and Experimental Design

**Figure 5:**
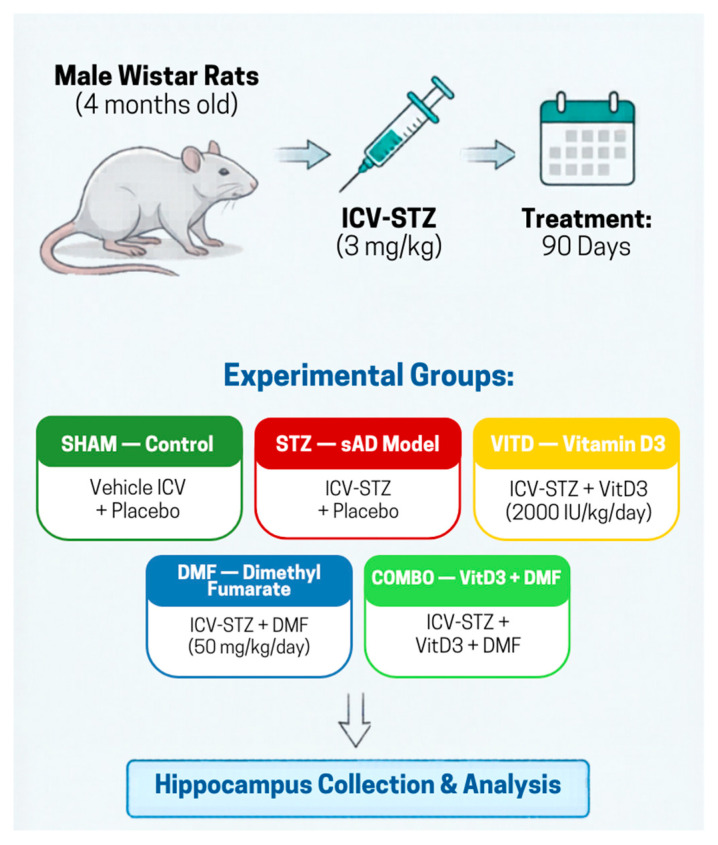
Study design. Male Wistar rats were assigned to five experimental groups: SHAM, STZ, VITD, DMF, and COMBO. The sporadic Alzheimer’s disease-like model was induced by intracerebroventricular streptozotocin (ICV-STZ; 3 mg/kg), followed by 90 days of treatment. After completion of the protocol, hippocampal tissue was collected for biochemical analyses. Created by the authors using Canva (Canva Pty Ltd., Sydney, Australia), under the Canva Content License Agreement.

Male Wistar rats aged 4 months were used in this study. Their initial body mass was 295.0 ± 36.1 g, and their final body mass at euthanasia was 428.2 ± 58.0 g. Animals were housed under standard conditions (22 °C, 55% humidity) with access to food and water ad libitum, under a 12 h light/dark cycle. All procedures involving animals were approved by the Local Ethical Committee for the Care and Use of Laboratory Animals in Bydgoszcz, Poland (approval No. 13/2022). Animals were randomly assigned to the following experimental groups: control (SHAM), sAD model with streptozotocin treatment (STZ), sAD model treated with vitamin D_3_ (VITD), sAD model treated with dimethyl fumarate (DMF), and sAD treated with both vitamin D_3_ and DMF (COMBO). The experimental design, animal handling, STZ administration, and treatment protocols were described in detail in our previous publication [16]. The present study represents an independent and complementary analysis of molecular parameters obtained from the same experimental cohort. Previously published behavioral data were not used as primary outcomes in the present study but were included only for exploratory correlation analyses with selected biochemical endpoints. A total of 50 male Wistar rats were initially included (*n* = 10 per group). Biochemical analyses were conducted on a subset of animals (*n* = 4 for WB and *n* = 6 for enzyme activity), depending on tissue availability and specific experimental requirements. No additional animals were used for this study. The present work represents a follow-up analysis focused on biochemical endpoints assessed in material obtained from that experiment (Figure 5).

### 4.2. Induction of the STZ Model and Treatment Administration

The sAD-like model was induced by intracerebroventricular (ICV) administration of streptozotocin (STZ) at a dose of 3 mg/kg, as described previously. Animals in the SHAM group received an equivalent volume of vehicle. Following induction of the ICV-STZ model, animals were treated with vitamin D_3_ (VitD_3_) (2000 IU/kg), DMF (50 mg/kg), or their combination, according to the protocol described in our previous publication. All treatments were administered orally once daily for 90 consecutive days. Animals assigned to the COMBO group received both compounds at the same doses as in the respective monotherapy groups. Detailed information regarding formulation, vehicle, and timing of administration was reported previously [16].

### 4.3. Tissue Collection and Sample Preparation

At the end of the experiment, animals were euthanized, and the brain tissue was collected. The hippocampus was dissected on ice, frozen, and stored at −80 °C until analysis. Tissue samples for enzymatic activity measurements were homogenized (4% *w*/*v*) in lysis buffer composed of 50 mM Tris–HCl, 150 mM NaCl, 1 mM EDTA, and 0.5 mM DTT containing 0.2% protease inhibitor cocktail (P834; Sigma-Aldrich, St. Louis, MO, USA), and the homogenates were centrifuged at 750× *g* for 10 min at 4 °C. For Western blot (WB) analysis, hippocampus tissue was homogenized (12% *w*/*v*) in Pierce™ RIPA buffer (Cat. 89901; Thermo Scientific™, Waltham, MA, USA) with EDTA-free Protease Inhibitor Cocktail (Cat. 04693159001; Roche, Basel, Switzerland) and PhosSTOP™ phosphatase inhibitors (Cat. 04906837001; Roche, Basel, Switzerland). The homogenates were centrifuged at 12,000× *g* for 10 min at 4 °C. Total protein concentration in both fractions was quantified using the Pierce™ BCA Protein Assay Kit (Cat. 23250; Thermo Scientific™, Waltham, USA) according to the manufacturer’s instructions.

### 4.4. Western Blot Analysis

Protein samples were separated by SDS-PAGE on [10%] gels and transferred onto PVDF membranes. Membranes were blocked with 5% non-fat milk in TBST and incubated overnight at 4 °C with primary antibodies against phospho-TrkB (Tyr816) (Cat. ABN1381; Sigma-Aldrich, St. Louis, USA), Akt (Cat. C67E7; Cell Signaling, Danvers, MA, USA), phospho-Akt (Thr308) (Cat. D25E6; Cell Signaling, Danvers, USA), IGF-1 (Cat. ab9572; Abcam, Cambridge, UK), BDNF (Cat. ab108319; Abcam, Cambridge, UK), and VDR (Cat. STJII3332; St. Johns Laboratory, London, UK). After incubation with HRP-conjugated secondary antibodies, immunoreactive bands were visualized using an enhanced chemiluminescence detection system. Band intensities were quantified densitometrically using Image Lab Software (Bio-Rad Laboratories, Inc., Hercules, CA, USA). All target proteins, including proBDNF, mature BDNF, VDR, IGF-1, phosphorylated TrkB (pTrkB, Tyr816), total Akt, and phosphorylated Akt (pAkt, Thr308), were normalized to the total protein load using stain-free technology. For Akt signaling, the pAkt/Akt ratio was additionally calculated on the basis of stain-free-normalized values.

### 4.5. Enzyme Activity Assays

Citrate synthase (CS) activity was measured at 37 °C in duplicate using the method described in [12]. Briefly, 2.5 μL of homogenate (4%) was incubated for 2 min in 165 μL of assay buffer (50 mM Tris-HCl, 1 mM EDTA, 0.01% Triton-X100, pH 7.8) supplemented with 20 μL of freshly made DTNB (10 mM) and 2 μL acetyl-CoA (50 mM). The reaction was initiated by adding 2 μL of freshly prepared oxaloacetic acid (10 mM). Absorbance was measured at 412 nm using a Beckman Coulter DTX 880 Multimode Detector (Beckman Coulter, Inc., Brea, CA, USA) The activity was expressed in μmol/min/mg protein.

Glutathione peroxidase (GPx) activity was measured at 30 °C in duplicate using a modified coupled assay as described in [30]. Briefly, 20 μL of 4% tissue homogenate was mixed with 40 μL of 10 mM potassium phosphate buffer (pH 7.2) containing 10 μL of GSH (10 mM), 10 μL of glutathione reductase (10 U/mL), and 10 μL of NADPH (5 mM). The reaction was initiated by the addition of 10 μL of freshly prepared cumene hydroperoxide (5 mM). Absorbance was monitored at 340 nm using a Beckman Coulter DTX 880 Multimode Detector (Beckman Coulter, Inc., Brea, USA), and GPx activity was calculated from the rate of NADPH oxidation and expressed as nmol of NADPH oxidized per minute per milligram of protein.

### 4.6. Statistical Analysis

Statistical analysis was performed using GraphPad Prism (version 9.0.2, Software, San Diego, CA, USA). Data are presented as mean ± standard deviation (SD). The normality of data distribution was assessed using the Shapiro–Wilk test, and homogeneity of variances was evaluated using the Brown–Forsythe test. For comparisons among multiple experimental groups, one-way ANOVA was applied, followed by Tukey’s multiple comparisons post hoc test. Differences were considered statistically significant at *p* < 0.05.

## 5. Conclusions

In conclusion, this study showed that STZ-induced neurodegeneration is associated with disturbances in selected neurotrophic-related parameters, characterized mainly by a reduced mature BDNF/proBDNF ratio and trend toward lower mature BDNF content, without robust changes in Akt-related downstream signaling. These molecular alterations occur in the context of redox imbalance and in the absence of significant changes in selected mitochondrial enzymes, suggesting that the observed biochemical profile may be more closely linked to redox-related functional disturbances than to overt structural mitochondrial damage. Combined Vit D_3_ and DMF treatment increased mature BDNF content and showed a trend toward improvement of the mature BDNF/proBDNF ratio, indicating partial modulation of selected aspects of this signaling. Overall, these findings support the view that targeting neurotrophic and redox-related mechanisms may represent a relevant strategy for limiting STZ-induced neurodegenerative changes. However, the protective efficacy of the combined treatment appears partial and requires further investigation.

## Data Availability

The original contributions presented in this study are included in the article/Supplementary Material. Further inquiries can be directed to the corresponding author(s). The data presented in this study are available within the article and Supplementary Materials. Uncropped Western blot images are attached to the manuscript submission. Additional raw data supporting the findings of this study are available from the corresponding author upon reasonable request.

## Supplementary Materials

The following supporting information can be downloaded at: https://www.mdpi.com/article/10.3390/ijms27114940/s1.
