# Supplementary material for: Vitamin D3 and Dimethyl Fumarate Partially Restore Neurotrophic Signaling Without Altering Mitochondrial Integrity in the STZ-Induced Model of Sporadic AD
Source: Int J Mol Sci. 2026 May 29;27(11):4940. doi: 10.3390/ijms27114940 (PMC13256348; doi:10.3390/ijms27114940)
Supplement: Supplementary file 1 [file ijms-27-04940-s001.zip › ijms-4303276-supplementary.pdf]

Supplementary Materials

This supplementary material contains full-length uncropped Western blot images, corresponding stain-free membrane images used for total protein normalization, exploratory OGDH data, and source data for the kinetic assays presented in the main manuscript.

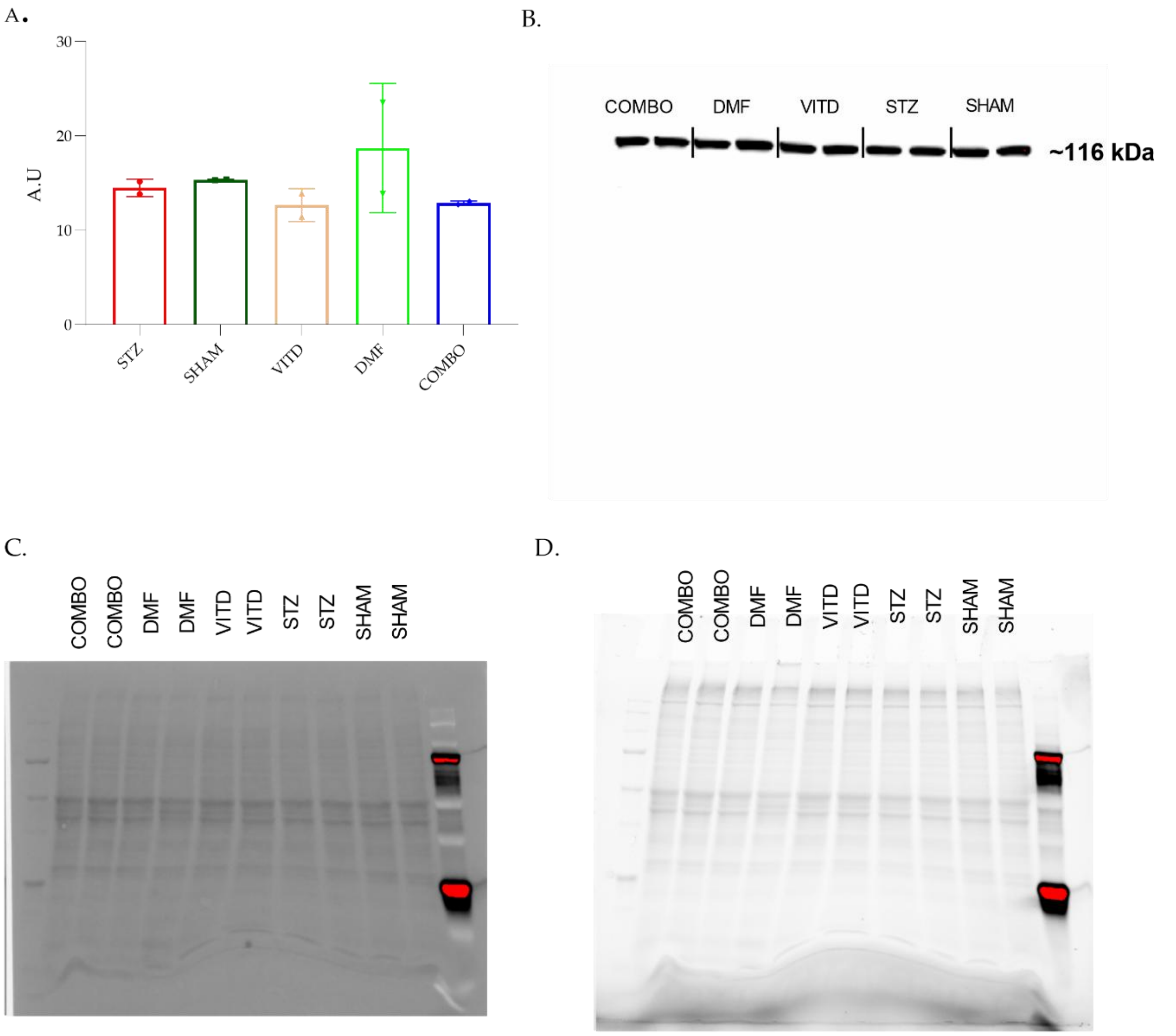

Figure S1. Exploratory OGDH analysis (n = 2). **A**. Densitometric quantification of OGDH normalized to total protein. **B**. Representative uncropped chemiluminescence image showing the OGDH-immunoreactive band at ~116 kDa. **C**. Representative stain-free blot used for total protein normalization. **D**. Corresponding stain-free gels. Due to the limited sample size (n = 2), these data are presented as exploratory and should be interpreted with caution. An exploratory analysis of OGDH protein content was performed; however, no clear group-dependent pattern was observed. Given the limited sample size (n = 2), these data are presented only as supplementary material and were not used for statistical analysis.

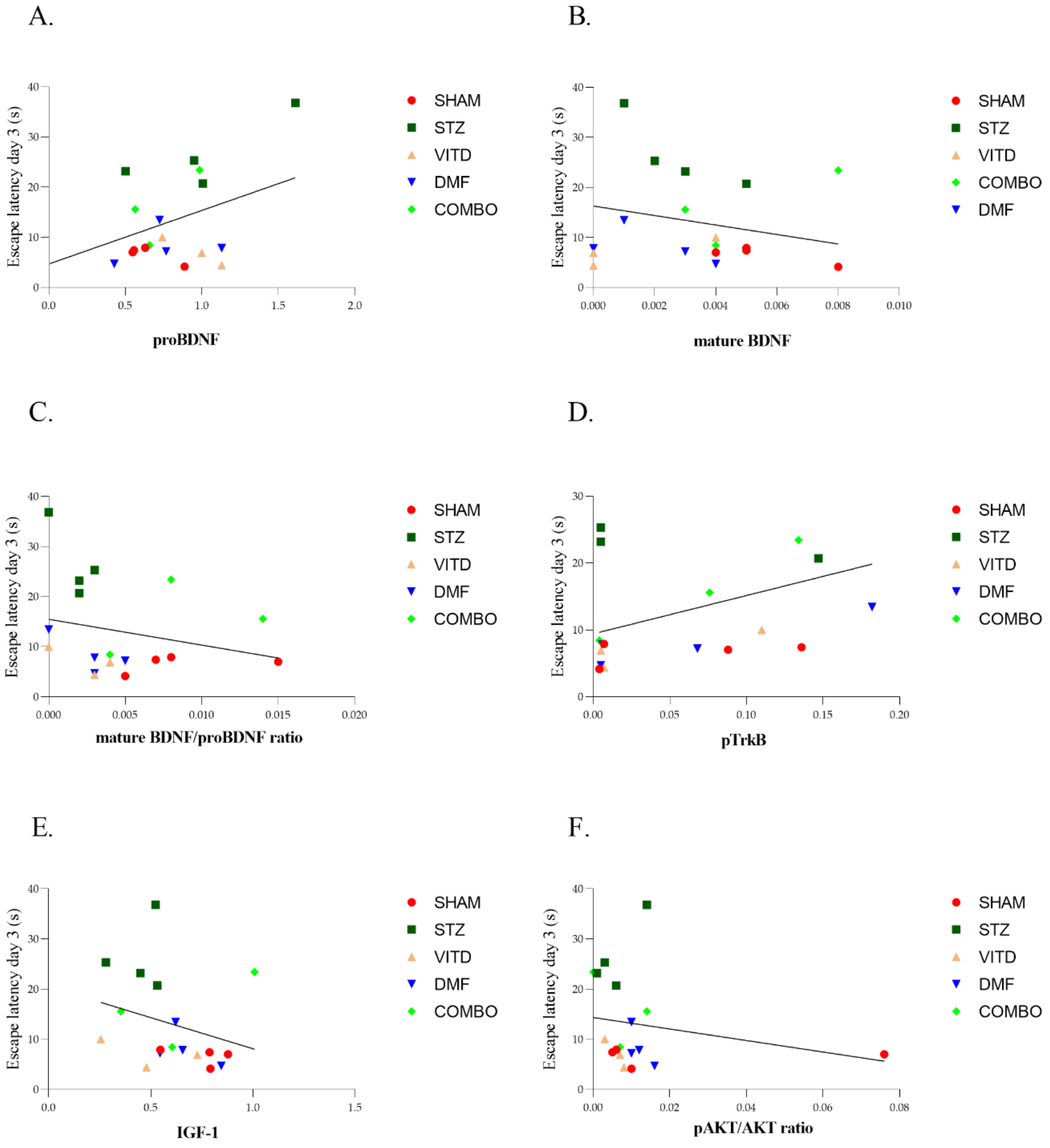

Figure S2. Spearman correlation analysis between hippocampal protein content and Morris water maze performance parameters measured on day 3. **A**. proBDNF vs. escape latency day 3 (r = 0.1950, p = 0.3970), **B**. mature BDNF vs. escape latency day 3 (r = −0.3401, p = 0.1673), **C**. mature BDNF/proBDNF ratio vs. escape latency day 3 (r = −0.3250, p = 0.188, **D**. pTRKB vs. escape latency day 3 (r = 0.4029, p = 0.0973), **E**. IGF-1 vs. escape latency day 3 (r = −0.4572, p = 0.0565), **F**. pAkt/Akt ratio vs. escape latency day 3 (r = −0.4052, p = 0.0953).
